# Case Report: A modified Karapandzic flap with bilateral V–Y advancement for reconstruction of a large full-thickness lower lip defect after squamous cell carcinoma excision

**DOI:** 10.3389/fsurg.2026.1984631

**Published:** 2026-10-28

**Authors:** Sang Il Seok, Soo Yeon Lim, In Chang Koh

**Affiliations:** Department of Plastic and Reconstructive Surgery, Myunggok Medical Research Center, Konyang University Hospital, University of Konyang College of Medicine, Daejeon, Republic of Korea

**Keywords:** case report, Karapandzic flap, lower lip reconstruction, squamous cell carcinoma, V–Y advancement flap

## Abstract

**Purpose:**

Reconstruction of full-thickness lower lip defects involving more than two-thirds of the vermilion remains surgically challenging. The conventional Karapandzic flap is a labial artery–based musculomucocutaneous flap requiring continuous incisions extending from the labiolabial crease through the labiomandibular fold to the mental crease, often resulting in extensive scarring. Moreover, this technique requires meticulous identification and preservation of the labial artery and perioral nerves, leading to greater technical demands and prolonged operative time, particularly for less experienced surgeons. Herein, we introduce a modified Karapandzic flap technique using bilateral V–Y advancement flaps for lower lip reconstruction following squamous cell carcinoma (SCC) excision.

**Methods:**

A 74-year-old man presented with SCC of the lower lip. The lesion measured 4.3 × 1.8 cm, and a wedge-shaped wide excision with a 5-mm surgical margin was performed. The resulting full-thickness defect measured 5.3 × 2.3 cm, involving more than two-thirds of the lower lip. Instead of extending incisions across the entire lower lip, bilateral V–Y advancement flaps were designed on both lateral aspects and elevated with partial release of the depressor anguli oris muscle to allow medial mobilization.

**Results:**

Complete flap survival and satisfactory wound healing were observed without major complications. At approximately four months, oral competence was maintained according to the patient, without liquid leakage, drooling, or food spillage, and without restrictions on food types. During maximal voluntary mouth opening, the intercommissural distance was 2.5 cm, the vertical oral aperture was 4 cm, and the distance between externally marked reference points corresponding to the palpated upper and lower incisal positions was 6 cm. Two-point discrimination was 5 mm at the central and bilateral lower lip sites. Postoperative microstomia resulted in reduced oral aperture, for which secondary commissuroplasty had been anticipated as part of a staged reconstructive plan.

**Conclusion:**

Bilateral V–Y advancement based on the modified Karapandzic concept may provide a reconstructive option for selected extensive full-thickness lower lip defects without formal neurovascular dissection. Although flap survival and oral competence were maintained in this case, postoperative microstomia and the anticipated need for secondary commissuroplasty should be considered during surgical planning.

## 1 Introduction

The lower lip significantly contributes to oral competence, speech articulation, facial expressions, and facial aesthetics (1, 2). Reconstruction of large full-thickness lower lip defects remains one of the most challenging procedures in facial reconstruction because both functional and aesthetic restorations must be achieved simultaneously (1, 2). Several reconstructive options have been described according to the defect size, including primary closure, local flaps (such as the Abbe flap, Estlander flap, Bernard–Webster flap, and Karapandzic flap), and free-tissue transfer (1–3). Recent reconstructive algorithms recommend primary closure for defects involving less than one-third of the lower lip, whereas larger defects often require local flap reconstruction (1). Defects involving more than two-thirds of the lower lip generally require more complex reconstructive procedures, such as the Karapandzic flap, Bernard–Webster flap, or free-tissue transfer (1–3). Among these techniques, the Karapandzic flap remains a widely accepted option because it preserves the neurovascular supply and continuity of the orbicularis oris (4–6). Preservation of the orbicularis oris sphincter allows maintenance of oral competence, speech, and sensory function (4, 5, 7). However, conventional Karapandzic reconstruction requires extensive circumoral incision and meticulous neurovascular dissection (5, 6). Furthermore, postoperative microstomia remains a well-recognized limitation of this technique (7). Herein, we present a case of successful reconstruction of a large full-thickness lower lip defect following squamous cell carcinoma (SCC) excision using bilateral V–Y advancement musculocutaneous flaps based on the modified Karapandzic concept.

## 2 Case description

### 2.1 Clinical presentation and findings

A 74-year-old man presented on July 14, 2025, with an ulcerative lesion involving the central lower lip. Physical examination revealed a 4.3 × 1.8 cm exophytic mass involving both the vermilion and cutaneous portions of the lower lip (Figure 1). Histopathologic evaluation confirmed keratoacanthoma-type SCC. Surgical excision and immediate reconstruction were planned.

**Figure 1:**
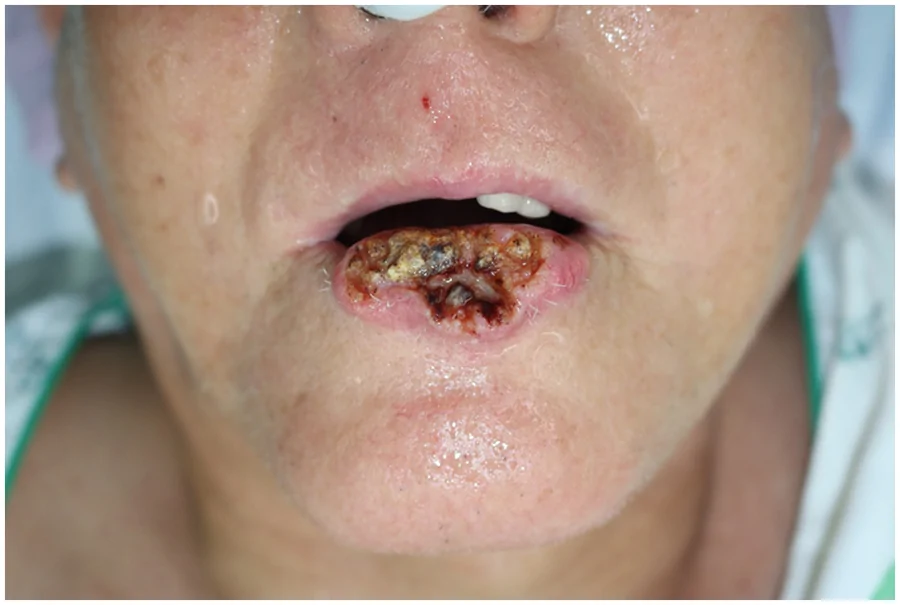
Preoperative clinical photograph demonstrating a keratoacanthoma-type squamous cell carcinoma involving the central lower lip.

### 2.2 Diagnostic assessment

Histopathologic evaluation of the lower lip lesion confirmed keratoacanthoma-type SCC. Intraoperative frozen-section analysis demonstrated tumor-free margins, permitting immediate reconstruction. Final histopathological examination confirmed SCC (pT1) with negative surgical margins. Postoperative radiotherapy and chemotherapy were not administered. Regional nodal and distant metastatic categories were not established in the available case information; therefore, an overall pathologic stage group is not assigned.

### 2.3 Therapeutic intervention

On September 18, 2025, the patient underwent surgical excision and immediate lower lip reconstruction under general anesthesia. A circular skin marking was made approximately 5 mm beyond the clinically visible tumor margin. Full-thickness excision was performed through the skin, orbicularis oris muscle, and oral mucosa. The lesion was removed *en bloc*, resulting in a through-and-through lower lip defect measuring 5.3 × 2.3 cm and involving more than two-thirds of the lower lip width (Figures 2A,B). Hemostasis was achieved using electrocautery. Following confirmation of tumor-free margins by frozen-section analysis, reconstruction was performed using a modified Karapandzic flap. Bilateral V–Y advancement flaps were designed along the nasolabial folds and labiomandibular creases. The incisions were carried through the subcutaneous tissue, and partial release of the depressor anguli oris muscles was performed bilaterally to facilitate flap advancement (Figures 2C,D). Unlike the conventional Karapandzic technique, formal identification and dissection of the labial arteries and perioral nerve branches were not required (2, 4). The bilateral musculocutaneous flaps were mobilized and advanced medially while preserving the surrounding neurovascular network. The orbicularis oris muscle was carefully reapproximated to restore sphincter continuity. The oral mucosa and skin were closed in layers, and dog-ear correction was performed to improve contour symmetry. The reconstruction was completed using a simplified flap elevation technique that avoided formal neurovascular dissection. Postoperative reduction of oral aperture was anticipated before the initial reconstruction. The possibility of microstomia and a planned second-stage commissuroplasty were discussed with the patient as part of the staged reconstructive strategy.

**Figure 2:**
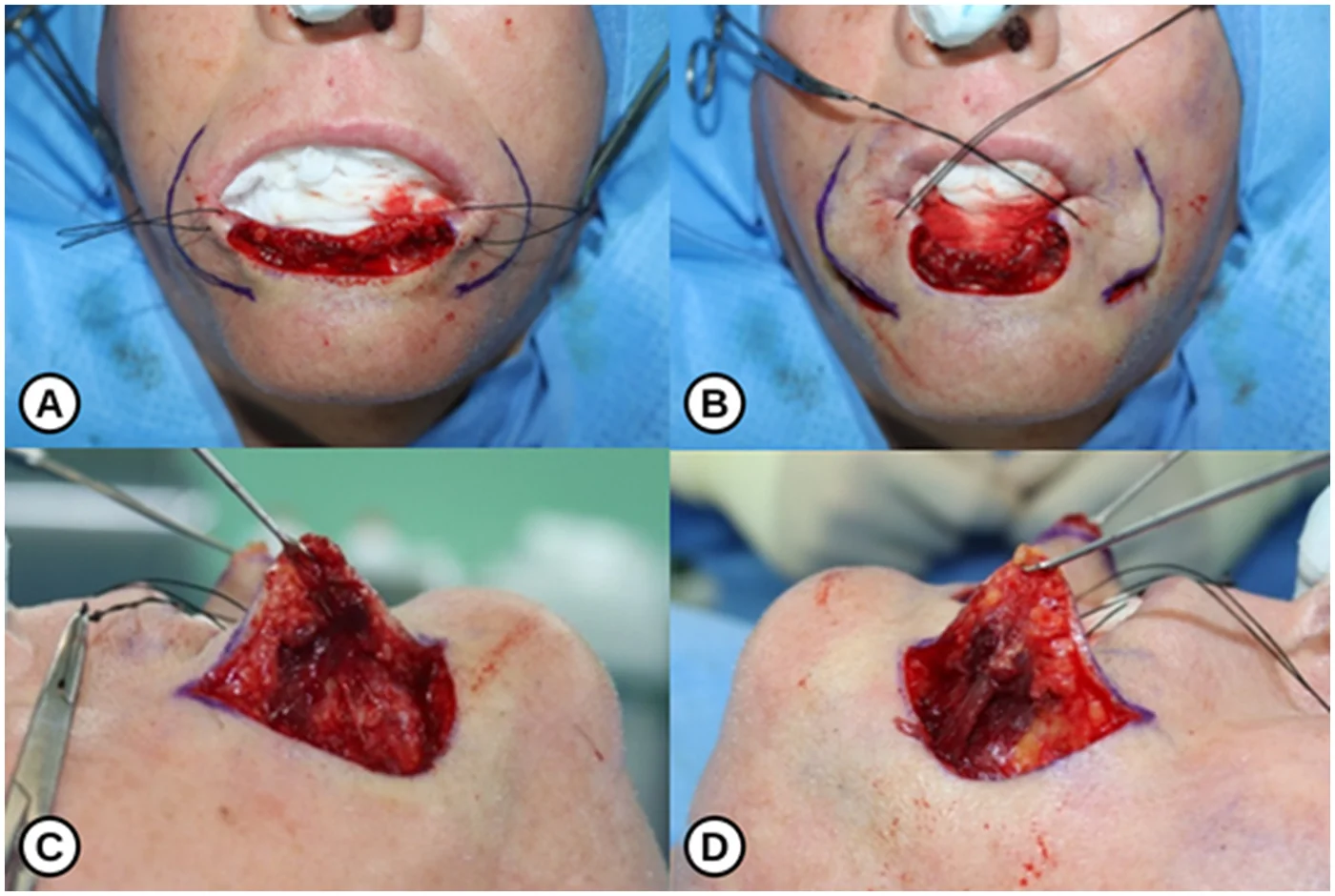
Intraoperative findings. **(A,B)** Full-thickness lower lip defect measuring 5.3 × 2.3 cm after wide excision with a 5-mm safety margin. **(C,D)** Reconstruction using bilateral V–Y advancement musculocutaneous flaps with partial release of the depressor anguli oris muscles.

### 2.4 Follow-up and outcomes

Postoperatively, flap perfusion remained satisfactory without evidence of vascular compromise. Mild tension at the central closure site was observed immediately after reconstruction, without ischemia or dehiscence (Figure 3A). Partial sutures were removed on September 26, 2025 (postoperative day 8), and the remaining sutures on September 30, 2025 (postoperative day 12). There was no infection, hematoma, flap necrosis, or wound breakdown. At the January 9, 2026 follow-up, approximately four months after the initial reconstruction and before secondary commissuroplasty, the reconstructed lip demonstrated stable healing and satisfactory contour (Figure 3B). During maximal voluntary mouth opening, the intercommissural distance was 2.5 cm and the vertical oral aperture, measured between the upper and lower lip margins, was 4 cm (Figure 3C). The positions of the upper and lower incisors were palpated and transferred to external skin markings; the distance between these external reference points was 6 cm. Because this was an indirect external landmark-based measurement, it should not be interpreted as a directly measured standard interincisal distance. Two-point discrimination testing performed on the same date showed a threshold of 5 mm at the central portion of the reconstructed lower lip and at both lateral lower lip sites. The patient reported preserved liquid retention without leakage, no drooling or food spillage, and no restriction in the types of food consumed. However, a reduced oral aperture consistent with postoperative microstomia was present. Secondary commissuroplasty had been anticipated preoperatively as part of a staged approach. No standardized lip-pursing or speech-movement photographs were obtained. No clinical evidence of local tumor recurrence was identified at this follow-up.

**Figure 3:**
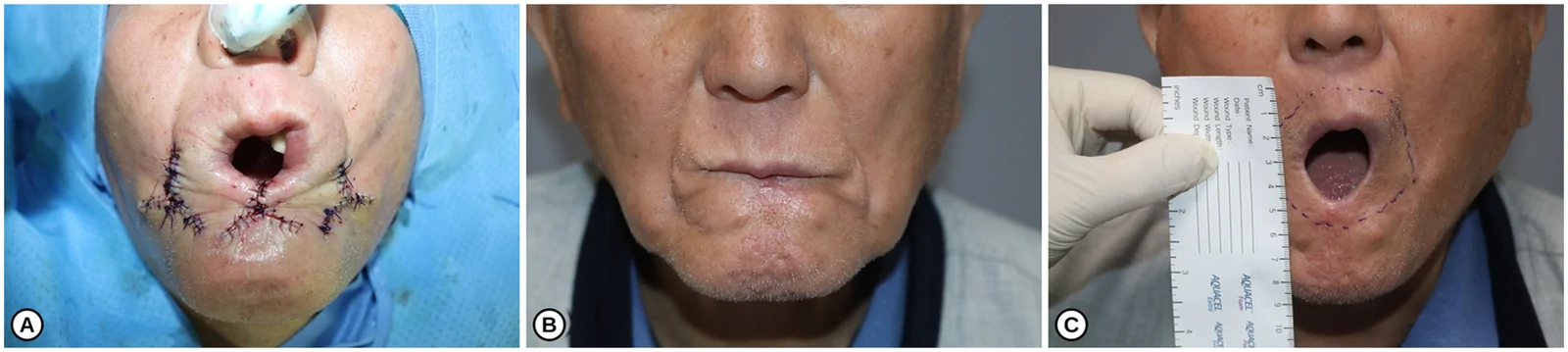
Postoperative outcomes. **(A)** Immediate postoperative appearance after the initial reconstruction. **(B)** Appearance on January 9, 2026, approximately four months after reconstruction and before secondary commissuroplasty, demonstrating stable healing and lip contour. **(C)** Maximal voluntary mouth opening at the same follow-up. The vertical oral aperture was 4 cm. The purple external markings indicate palpated upper and lower incisor positions transferred to the skin; the distance between these marked reference points was 6 cm and represents an indirect external estimate rather than a direct interincisal measurement. The intercommissural distance was 2.5 cm (not directly demonstrated by this image).

The study was approved by the Institutional Review Board (IRB) of Konyang University Hospital (IRB No. 2026-08-020). Written informed consent was obtained from the patient for publication of this case report and any potentially identifiable images or data included in this article. This case report was prepared in accordance with the CARE guidelines.

## 3 Timeline

A chronological timeline of the patient’s presentation, surgical intervention, and postoperative follow-up is provided in Table 1.

**Table 1:** Chronological timeline of the patient's clinical course.

Date	Clinical event	Findings and management
July 14, 2025	Initial presentation	A 74-year-old man presented with an ulcerative exophytic lesion measuring 4.3 × 1.8 cm involving the vermilion and cutaneous portions of the central lower lip.
September 18, 2025	Surgical excision and reconstruction	Full-thickness excision with an approximately 5-mm surgical margin resulted in a 5.3 × 2.3 cm through-and-through defect involving more than two-thirds of the lower lip. Frozen-section analysis confirmed tumor-free margins. Reconstruction was performed using bilateral V–Y advancement musculocutaneous flaps with partial release of the depressor anguli oris muscles.
September 26, 2025 (POD 8)	Postoperative follow-up	Partial suture removal was performed. The wound was healing satisfactorily without flap-related complications.
September 30, 2025 (POD 12)	Postoperative follow-up	Complete suture removal was performed. Wound healing remained uneventful.
January 9, 2026	Follow-up	Stable healing and lip contour. During maximal voluntary opening, intercommissural distance 2.5 cm and vertical oral aperture 4 cm; distance between externally marked palpated incisor positions 6 cm (indirect estimate). Two-point discrimination 5 mm at central and bilateral lower lip sites. Patient reported preserved liquid retention without drooling or food spillage and no restriction on food types. Microstomia remained; secondary commissuroplasty had been anticipated in the staged plan. No local recurrence observed at this visit.

POD, Postoperative day.

## 4 Discussion

Reconstruction of extensive full-thickness lower lip defects requires balancing oral competence, adequate aperture, sensation, and appearance (1–3). The conventional Karapandzic flap maintains orbicularis oris continuity and its neurovascular supply but can cause microstomia and conspicuous circumoral scars (4–8). In the present case, bilateral V–Y advancement with partial release of the depressor anguli oris muscles allowed medial mobilization without formal labial artery or perioral nerve dissection. This modification may reduce the extent of circumoral dissection; however, preservation of function cannot be attributed solely to avoidance of formal neurovascular dissection on the basis of one case. In contrast, Bernard–Webster reconstruction may recruit additional tissue for broad defects but can alter sphincter continuity, whereas free-tissue transfer is appropriate for selected very extensive defects or insufficient local tissue, at the expense of greater procedural complexity (1–3). Choice of reconstruction must be individualized according to residual lip tissue, defect configuration, comorbidities, and the patient's priorities. For defects exceeding two-thirds of the lower lip, a modified local advancement strategy should not be assumed to prevent microstomia or to replace other reconstructive options in all patients.

At the four-month follow-up, the patient reported preserved liquid retention without leakage, no drooling or food spillage, and no restrictions on types of food consumed. These patient-reported findings support maintained oral competence but do not establish normal speech or unrestricted oral aperture. Objective measurements documented a 2.5-cm intercommissural distance and a 4-cm vertical oral aperture during maximal opening. The 6-cm distance between externally marked palpated incisor positions is an indirect estimate, not a direct interincisal measurement. Two-point discrimination was 5 mm centrally and bilaterally, demonstrating discriminatory sensation at the tested sites; without preoperative or serial testing, this cannot establish sensory improvement or superiority to conventional reconstruction. Microstomia remained an important consequence despite satisfactory flap survival. Its likelihood was anticipated before surgery, and a subsequent commissuroplasty was included in the planned staged treatment. Thus, the value of this approach is best considered in the context of possible secondary correction rather than as a single-stage solution for all large lower lip defects.

The first operation prioritized restoration of lower lip continuity and oral competence, while definitive enlargement of the oral aperture was deferred to a second stage. The present case should therefore not be interpreted as evidence that bilateral V–Y advancement alone reliably achieves adequate oral aperture in all defects exceeding two-thirds of the lower lip. The need for additional release or alternative reconstruction should be assessed according to defect extent, tissue mobility, and functional requirements.

This report has several limitations. It describes a single patient with approximately four months of follow-up after the initial reconstruction, limiting generalizability and assessment of late scar contracture or oncologic outcomes. Preoperative oral-aperture measurements and standardized functional testing were unavailable, preventing direct before-and-after comparisons. The external landmark-based estimate of incisal separation is not equivalent to a standardized direct interincisal measurement. Although postoperative two-point discrimination was documented, Semmes–Weinstein monofilament testing and serial sensory measurements were not performed. Dynamic photographs documenting lip pursing or speech-related movement were not obtained, and no standardized speech or oral-competence testing was performed; relevant functional findings were patient-reported. The anticipated second-stage commissuroplasty also limits interpretation of the initial procedure as a definitive single-stage reconstruction. Larger studies with standardized functional and sensory measurements and longer follow-up are warranted.

In summary, bilateral V–Y advancement using a modified Karapandzic concept achieved viable coverage of a large full-thickness lower lip defect and maintained patient-reported oral competence. Objective postoperative assessment demonstrated preserved discriminatory sensation but also reduced oral aperture. The potential need for staged commissuroplasty should be incorporated into preoperative counseling when considering this technique for selected extensive defects.

## 5 Patient perspective

At follow-up, the patient reported being able to retain liquids without leakage, with no drooling or food spillage and no restrictions on the types of food consumed. Nevertheless, the reduced oral aperture after the initial reconstruction remained a concern addressed by the preoperatively anticipated staged reconstructive plan. These outcomes reflect the patient's reported experience; standardized speech or dynamic lip-movement assessments were not performed.

## Data Availability

The original contributions presented in the study are included in the article/Supplementary Material, further inquiries can be directed to the corresponding author.
