# Supplementary material for: Case Report: A modified Karapandzic flap with bilateral V–Y advancement for reconstruction of a large full-thickness lower lip defect after squamous cell carcinoma excision
Source: Front Surg. 2026 Oct 28;13:1984631. doi: 10.3389/fsurg.2026.1984631 (PMC13751597; doi:10.3389/fsurg.2026.1984631)
Supplement: Supplementary file 1 [file Datasheet1.pdf]

CARE Checklist – Frontiers in Surgery Case Report

Manuscript: Case Report: A Modified Karapandzic Flap with Bilateral V–Y Advancement for Reconstruction of a Large Full-Thickness Lower Lip Defect after Squamous Cell Carcinoma Excision

**Topic**	**Item**	**CARE checklist item**	**Reported on**
Title	1	Diagnosis/intervention and identification as case report	p. 1
Key Words	2	2–5 keywords including “case report”	p. 1
Abstract	3a	Uniqueness and contribution	p. 1–2
Abstract	3b	Symptoms / key clinical findings	p. 1
Abstract	3c	Diagnosis, intervention, outcomes	p. 1–2
Abstract	3d	Take-away conclusion	p. 2
Introduction	4	Background and literature	p. 2
Patient Information	5a	De-identified patient information	p. 2
Patient Information	5b	Primary concerns / symptoms	p. 2
Patient Information	5c	Medical, family, psychosocial history	Not reported
Patient Information	5d	Relevant past interventions	Not reported
Clinical Findings	6	Physical examination and findings	p. 2
Timeline	7	Important dates in chronological form	Table 1, p. 6–7; see also p. 4
Diagnostic Assessment	8a	Diagnostic methods	p. 2
Diagnostic Assessment	8b	Diagnostic challenges	Not reported
Diagnostic Assessment	8c	Diagnosis and differential diagnoses	p. 2
Diagnostic Assessment	8d	Prognostic characteristics	p. 2
Therapeutic Intervention	9a	Type of intervention	p. 2–3
Therapeutic Intervention	9b	Procedural details	p. 2–3
Therapeutic Intervention	9c	Changes to intervention, with reasons	Not applicable to index procedure; staged plan described pp. 2–3
Follow-up and Outcomes	10a	Clinician- and patient-assessed outcomes	p. 3
Follow-up and Outcomes	10b	Follow-up tests and results	p. 3
Follow-up and Outcomes	10c	Adherence / tolerability and assessment	Not formally assessed / not reported
Follow-up and Outcomes	10d	Adverse and unanticipated events	p. 3–4
Discussion	11a	Strengths and limitations	p. 4–5
Discussion	11b	Relevant literature	p. 4
Discussion	11c	Rationale for conclusions	p. 4–5
Discussion	11d	Take-away lessons	p. 5
Patient Perspective	12	Patient-reported experience	p. 5
Informed Consent	13	Patient consent	p. 3
